# Application of CT-based foundational artificial intelligence and radiomics models for prediction of survival for lung cancer patients treated on the NRG/RTOG 0617 clinical trial

**DOI:** 10.1093/bjro/tzae038

**Published:** 2024-11-06

**Authors:** Taman Upadhaya, Indrin J Chetty, Elizabeth M McKenzie, Hassan Bagher-Ebadian, Katelyn M Atkins

**Affiliations:** Department of Radiation Oncology, Cedars-Sinai Medical Center, Los Angeles, California, 90048, United States; Department of Radiation Oncology, Cedars-Sinai Medical Center, Los Angeles, California, 90048, United States; Department of Radiation Oncology, Cedars-Sinai Medical Center, Los Angeles, California, 90048, United States; Department of Radiation Oncology, Henry Ford Hospital, Detroit, Michigan, 48202, United States; Department of Radiation Oncology, Cedars-Sinai Medical Center, Los Angeles, California, 90048, United States

**Keywords:** radiomics, foundational model, prognosis, non-small cell lung cancer, machine learning, feature selection, cross-validation

## Abstract

**Objectives:**

To apply CT-based foundational artificial intelligence (AI) and radiomics models for predicting overall survival (OS) for patients with locally advanced non-small cell lung cancer (NSCLC).

**Methods:**

Data for 449 patients retrospectively treated on the NRG Oncology/Radiation Therapy Oncology Group (RTOG) 0617 clinical trial were analyzed. Foundational AI, radiomics, and clinical features were evaluated using univariate cox regression and correlational analyses to determine independent predictors of survival. Several models were fit using these predictors and model performance was evaluated using nested cross-validation and unseen independent test datasets via area under receiver-operator-characteristic curves, AUCs.

**Results:**

For all patients, the combined foundational AI and clinical models achieved AUCs of 0.67 for the Random Forest (RF) model. The combined radiomics and clinical models achieved RF AUCs of 0.66. In the low-dose arm, foundational AI alone achieved AUC of 0.67, while AUC for the ensemble radiomics and clinical models was 0.65 for the support vector machine (SVM). In the high-dose arm, AUC values were 0.67 for combined radiomics and clinical models and 0.66 for the foundational AI model.

**Conclusions:**

This study demonstrated encouraging results for application of foundational AI and radiomics models for prediction of outcomes. More research is warranted to understand the value of ensemble models toward improving performance via complementary information.

**Advances in knowledge:**

Using foundational AI and radiomics-based models we were able to identify significant signatures of outcomes for NSCLC patients retrospectively treated on a national cooperative group clinical trial. Associated models will be important for application toward prospective patients.

## Introduction

In radiation oncology, information from imaging datasets, such as PET, computed tomography (CT), MRI can be used to explore relationships between treatment regimens and outcomes to develop models that understand responses to treatment and better stratify treatment regimens.^1^ Artificial intelligence (AI) and machine learning (ML) algorithms have become popular toward establishing associations between outcomes and imaging datasets.^2^ Supervised ML methods are commonly used for outcomes prediction, utilizing feature extraction or deep learning techniques to model outcomes.^3–5^ A foundational model is an AI model built using a self-supervised learning technique on a vast amount of unlabeled data, enabling it to be applied to a wide range of downstream applications.^6^ By incorporating quantification methods, such as self-supervised foundational models that encode spatial information, complementary information can be integrated to enhance predictive accuracy. The lack of need for labeled training data facilitates training of foundational models on large scale image datasets. Recently, foundational models have shown significantly enhanced performance in both language and vision tasks, driving successes in applications such as ChatGPT, BERT, and CLIP for natural language processing (NLP), as well as SimCLR, YOLO, DINO, SAM, and DALLE for computer vision.^7^^,^^8^

Application of foundational models for 3D medical imaging is an emerging area of research. Examples include, MED-SAM used for 3D organ segmentation and BrainSegFounder used for neuroimage segmentation.^9^^,^^10^ Furthermore, BiomedGPT integrates multiple modalities such as radiographs, digital images, and text into a unified framework, enabling a diverse range of biomedical tasks.^11^ In the domain of medical image analysis leveraging a foundational model for downstream tasks helps address the challenge of both limited and unlabeled data for modeling. To date, the only known foundational model specifically for imaging biomarker was published by Pai et al^12^ which used a comprehensive dataset of 11 467 radiographic cancers on CT imaging for various clinically relevant tasks such as classifying lesion anatomy, creating a diagnostic biomarker to determine the malignancy of lung nodules, and developing a prognostic biomarker for non-small cell lung cancer (NSCLC) tumors.

Studies have shown that high-resolution medical images contain abundant meaningful information about shape, intensity, and texture that can be used to characterize the stochastic properties of tissues of interest. Radiomics refers to the high-throughput extraction of high-dimensional quantitative features from medical images available to the oncology imaging community.^13–15^ Radiomics holds promise toward characterization of cancers by analysis of shape, intensity, and texture of tumors and organs on medical images. The associated radiomics features represent signatures of disease and outcome. Radiomics has been clinically applied to screening, diagnosis, treatment, and follow-up.^16–22^ While there is extensive research on handcrafted radiomics features (intensity, texture, morphological) and deep radiomics (based on deep learning), research on foundational AI architectures, which can incorporate radiomics features for predicting oncologic outcomes, remains largely unexplored.^20^

In this study we aimed to retrospectively implement and evaluate the performance of foundational and hand-crafted radiomics-based models for prediction of 2-years overall survival (OS) outcomes for patients with locally advanced lung cancers treated on the NRG/RTOG 0617 clinical trial.^23^^,^^24^

## Methods

### Clinical cohort description

The NRG Oncology/Radiation Therapy Oncology Group (RTOG) 0617 dataset from the National Clinical Trials Network (NCTN) was utilized.^23^^,^^24^ All relevant pre-treatment non-contrast CT (slice thickness = 2.5-3 mm) images, RT plans, and clinical features such as Zubrod performance status, tumor histology, age, race, gender, smoking history, pneumonitis, esophagitis, chemotherapy, and RT dose groups (60 vs 74 Gy) were analyzed as per the NCTN dataset (Table 1).^23^^,^^25^ Additionally, dose statistics (dose volume histogram [DVH]) from heart, left anterior descending coronary artery, planning tumor volume (PTV), clinical tumor volume (CTV), ipsilateral lung, and esophagus normal tissues were included in the analysis.^26^ Survival time and vital status were used to estimate the 2-year OS. The 2-year time frame is a standard clinical endpoint for NSCLC patients because this period helps clinicians understand the effectiveness of current treatment protocols and provides a benchmark for comparing new therapies. Several other prediction models have used the 2-year survival endpoint.^27–30^ All analyses and conclusions in this manuscript are the sole responsibility of the authors and do not necessarily reflect the opinions or views of the clinical trial investigators, the NCTN, or the NCI.

**Table 1.** Patient, tumor, and treatment characteristics.^20^^,^^21^

Characteristic	All dose		High dose		Low dose	
	Training No. (%)	Testing No. (%)	Training No. (%)	Testing No. (%)	Training No. (%)	Testing No. (%)
Number of patient	278 (67%)	140 (33%)	184 (67%)	46 (33%)	150 (67%)	38 (33%)
Age at diagnosis, median (range)	64 (37-82)	64 (40-83)	65 (37-82)	60 (38-82)	64 (42-83)	63 (41-74)
Survival time (months), median (range)	23 (0-61)	23 (1-60)	24 (1-61)	24 (2-60)	20 (0-60)	17.5 (1-51)
2-year survival rate	49.5%	49.3%	53.9%	53.8%	44%	44.7%
Gender						
Male	171 (62%)	82 (59%)	111 (60%)	30 (65%)	91 (61%)	21 (55%)
Female	107 (38%)	58 (41%)	73 (40%)	16 (35%)	59 (39%)	17 (45%)
AJCC Stage (6th edition, 2002) IIIA, or N2/undetectable primary	185 (67%)	90 (64%)	126 (68%)	30 (65%)	95 (63%)	24 (63%)
IIIB, or N3/undetectable primary	93 (33%)	50 (36%)	58 (32%)	16 (35%)	55 (37%)	14 (37%)
Zubrod performance status						
Normal activity	169 (60%)	77 (55%)	110 (60%)	25 (54%)	91 (61%)	20 (53%)
Symptoms, but nearly fully ambulatory	109 (39%)	63 (45%)	74 (40%)	21 (46%)	59 (39%)	18 (47%)
Smoking history						
Non-smoker	15 (5%)	11 (8%)	11 (6%)	4 (9%)	11 (7%)	0 (0%)
Former light smoker	24 (9%)	14 (10%)	17 (9%)	5 (11%)	(7%)	5 (13%)
Former heavy smoker	93 (33%)	50 (36%)	68 (37%)	15 (33%)	50 (33%)	10 (26%)
Current smoker	133 (48%)	57 (57%)	75 (41%)	19 (41%)	74 (49%)	22 (58%)
Unknown	13 (5%)	8 (6%)	13 (7%)	3 (7%)	4 (3%)	1 (3%)
Chemotherapy (Cetuximab)						
Yes	124 (45%)	67 (48%)	82 (45%)	20 (43%)	70 (47%)	19 (50%)
No	154 (55%)	73 (52%)	102 (55%)	26 (57%)	80 (53%)	19 (50%)
Radiotherapy						
60 Gy	142 (51%)	88 (63%)	0 (0%)	0 (0%)	150 (100%)	36 (100%)
70 Gy	136 (49%)	52 (37%)	184 (100%)	46 (100%)	0 (0%)	0 (0%)
RT technique						
3D-CRT	148 (53%)	75 (54%)	98 (53%)	24 (52%)	83 (55%)	18 (47%)
IMRT	130 (47%)	65 (46%)	86 (47%)	22 (48%)	67 (45%)	20 (53%)
Histology						
Squamous cell carcinoma	121 (44%)	60 (43%)	77 (42%)	18 (39%)	73 (49%)	13 (34%)
Adenocarcinoma	106 (38%)	57 (41%)	75 (41%)	21 (46%)	54 (36%)	13 (34%)
Large cell undifferentiated	9 (3%)	2(1%)	4 (2%)	1 (1%)	5 (3%)	1 (3%)
Non-small cell lung cancer NOS	42 (15%)	21 (15%)	28 (15%)	6 (13%)	18 (12%)	11 (29%)
Race						
American Indian/Alaskan Native	0 (0%)	2 (1%)	1 (0.5%)	0 (0%)	1 (0.5%)	0 (0%)
Asian	6 (2%)	4 (3%)	6 (3%)	0 (0%)	3 (2%)	1 (3%)
Black or African American	25 (9%)	14 (10%)	15 (8%)	4 (9%)	17 (11%)	3 (8%)
Native Hawaiian/Other Pacific Islander	1 (0.35%)	0 (0%)	1 (0.5%)	0 (0%)	0 (0%)	0 (0%)
White	245 (88%)	117 (84%)	158 (86%)	41 (89%)	129 (86%)	34 (89%)
Unknown	1 (0.35%)	3 (2%)	3 (2%)	1 (2%)	0 (0%)	0 (0%)

### Foundational AI

**Figure 1.**
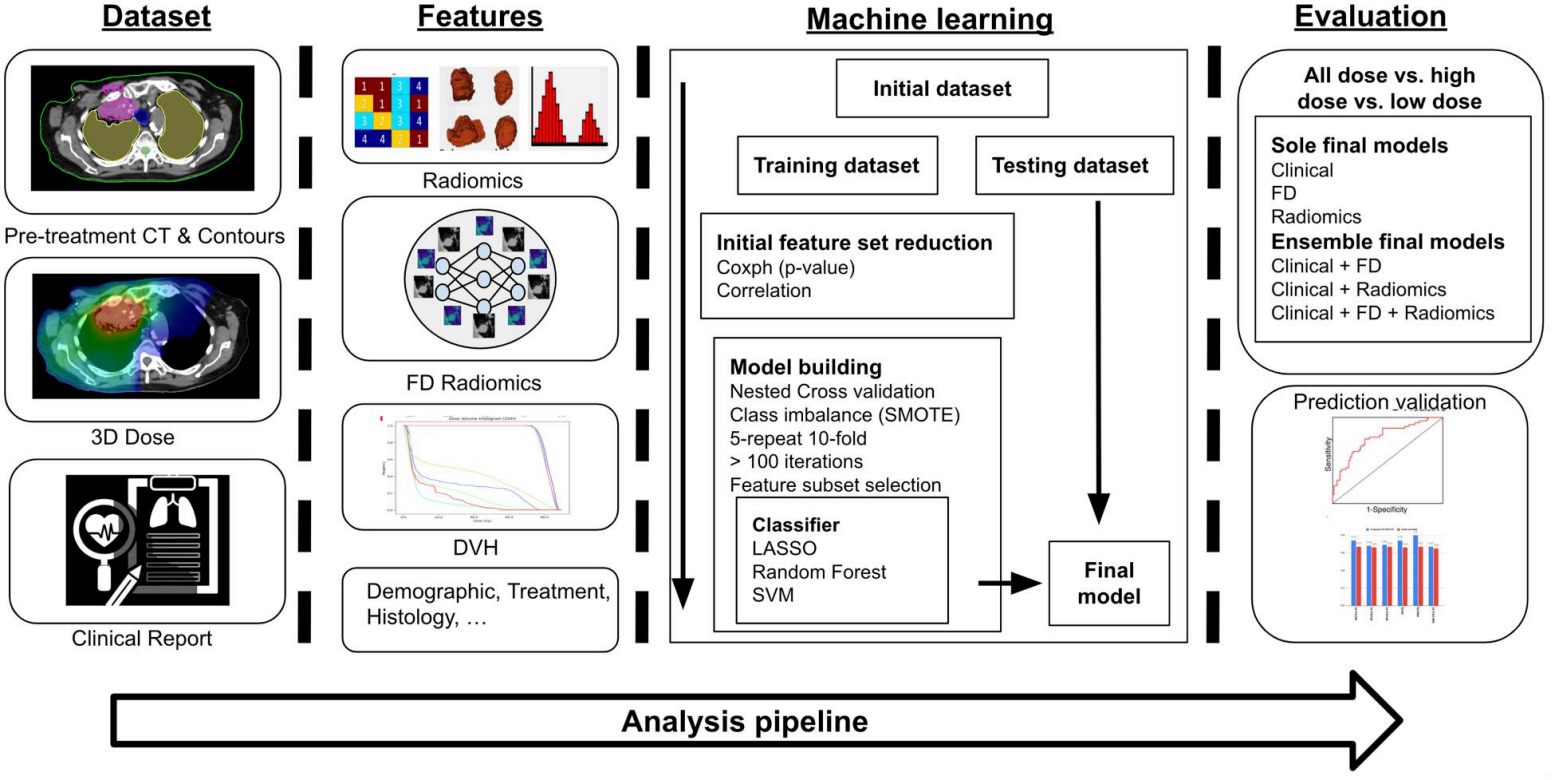
Summary of the various radiomics workflow: schematic steps includes collection of patient clinical information and image (CT and dose) dataset, extracting clinical, DVH, radiomics and foundational AI feature, feature selection, and machine learning model construction for the development of the predictive models.

We utilized a foundational model developed by Pai et al^12^ which was pre-trained on 11 467 using segments from tumors of different cancer types identified on CT imaging. This foundational model is a modified version of the original SimCLR framework, which was developed for medical imaging applications.^31^ It adopts the ResNet50 architecture^32^ as encoder with a contrastive loss function in a self-supervised manner to learn to identify positive pairs of 3D patches surrounding the lesion and negative pairs, randomly sampled 3D patches from the background.^12^^,^^33^ For our specific task, we modified the pretrained model by incorporating lesion-based clipping in the image transformation step to accommodate tumors of various sizes and multiple lesions within the region of interest (ROI). We recommend interested readers to consult the original paper for a deeper understanding.^12^^,^^33^ We used these ROIs to extract 4098 features and built models to predict the 2-year OS for the patient cohort (Figure 1).

### Radiomics features

Associated with the NRG/RTOG 0617 clinical trial, contours of the gross tumor volume (GTV) or tumor volume-of-interests (VOIs) defined on the planning CT images were analyzed. For each VOI, hand-crafted radiomics features were extracted using appropriate mathematical transformation algorithms. These included 29 morphological features, 43 intensity-based features (18 statistical features, 23 intensity histogram, 7 intensity-volume histogram, 2 local intensity features), and 94 textural features (25 grey level co-occurrence matrix [GLCM], 16 grey level run length matrix [GLRLM], 16 grey level size zone matrix [GLSZM], 21 neighboring grey level dependence matrix [NGLDM], 16 grey level distance zone matrix [NGLDM]), extracted in 3-D according to the guidelines defined by the image biomarker standardization initiative (IBSI).^15^^,^^32^ Intensity histogram and Textural features were computed according to 12 different combinations of the following parameters, a process defined as “feature optimization,”^34^ resulting in different “variants” for each feature: (1) isotropic voxel sizes of 1 and 2 mm^3^ (ie, images were first interpolated to these dimensions using linear interpolation), (2) fixed bin number (FBN) and fixed bin size (FBS) gray-level discretization, and (3) number of gray levels: 32, 64, and 128. A total of 1557 radiomics features were extracted from the tumor VOI’s.

### Feature dimensionality reduction and subsets selection

Feature selection and ranking was performed to reduce dimensionality by removing redundant features or those that were poorly correlated with the outcome. Feature selection in training data was performed using: (1) univariate cox regression (*P*-value ≤ .05) to identify significant features (2) correlation analysis (CC ≤ 0.7) to identify independent predictors of survival, and (3) model building using nested 5-repeat 10-fold cross-validation where the inner loop ranked the features according feature importance measure of algorithm and the outer loop identified the subsets of features with highest accuracy. For feature subset selection, we exploited importance measures as appropriate for each algorithm: the weighted magnitude vector for support vector machine (SVM),^35^ the total decrease in node impurities (Gini index) averaged over all trees for Random Forest (RF),^36^ and the coefficients ranked by magnitude for LASSO.^37^

### ML modeling

Predictive models, LASSO/GLM, RF, and SVM were trained. Pre-processing for removing zero-variance features and centering (mean of zero and standard deviation of one) was applied. Class imbalance was handled using the Synthetic Minority Over-sampling Technique (SMOTE^38^) and tuning of the parameters for SVM, RF, and GLM/LASSO were done using 5-repeat, 10-fold cross-validation using stratified random sampling. All independent predictors were rank based on the importance score from LASSO, RF, and SVM and then stepwise forward feature selection based on the previous ranking was used to select the subset of features minimizing the validation error. All model building steps were applied in training dataset and the final model for testing was chosen based on the highest area under the curve (AUC). For other technical details, we direct readers to our prior publications.^39^^,^^40^ Three distinct experimental setups were carried out in accordance with the different arms of the clinical trial: (1) all RTOG 0617 patients (*n* = 449) were included (both high [74 Gy] and low dose [60 Gy] treatment arms). After pre-processing, the sample size was split as follows: *n* = 278 (training) and *n* = 140 (testing); (2) patients in the high dose arm only with split: *n* = 184 (training), *n* = 46 (testing); and (3) patients in the low dose arm only with split: *n* = 150 (training), *n* = 38 (testing). For each of these cohorts, we trained separate models based on foundational AI, radiomics, and clinical factors associated with outcomes. Although the analysis has been done on pre-treatment (simulation) CT image datasets, where treatment delivered dose was not relevant, having a model corresponding to each dose arm enabled us to determine whether there were significant differences in radiomics features between low- and high-dose arms, prior to deliver of radiation.

To determine the potential for complimentary information to enhance model performance, we also developed ensemble models by combining the separate models based on foundational AI, radiomics, and clinical factors. Analysis was performed for the different algorithms, RF, SVM, and Lasso/GLM, resulting in a total of 54 different predictive models. Implementation was carried out using the *R* software (R Foundation for Statistical Computing, v4.3, Vienna, Austria) and the Caret module.^41^^,^^42^ Given that the CT image datasets were anonymized and did not include scanning protocols for the many different institutions contributing patients to the RTOG0617 clinical trial, we were not able to harmonize intensities following methods, such as ComBat.^43^

## Results

**Figure 2.**
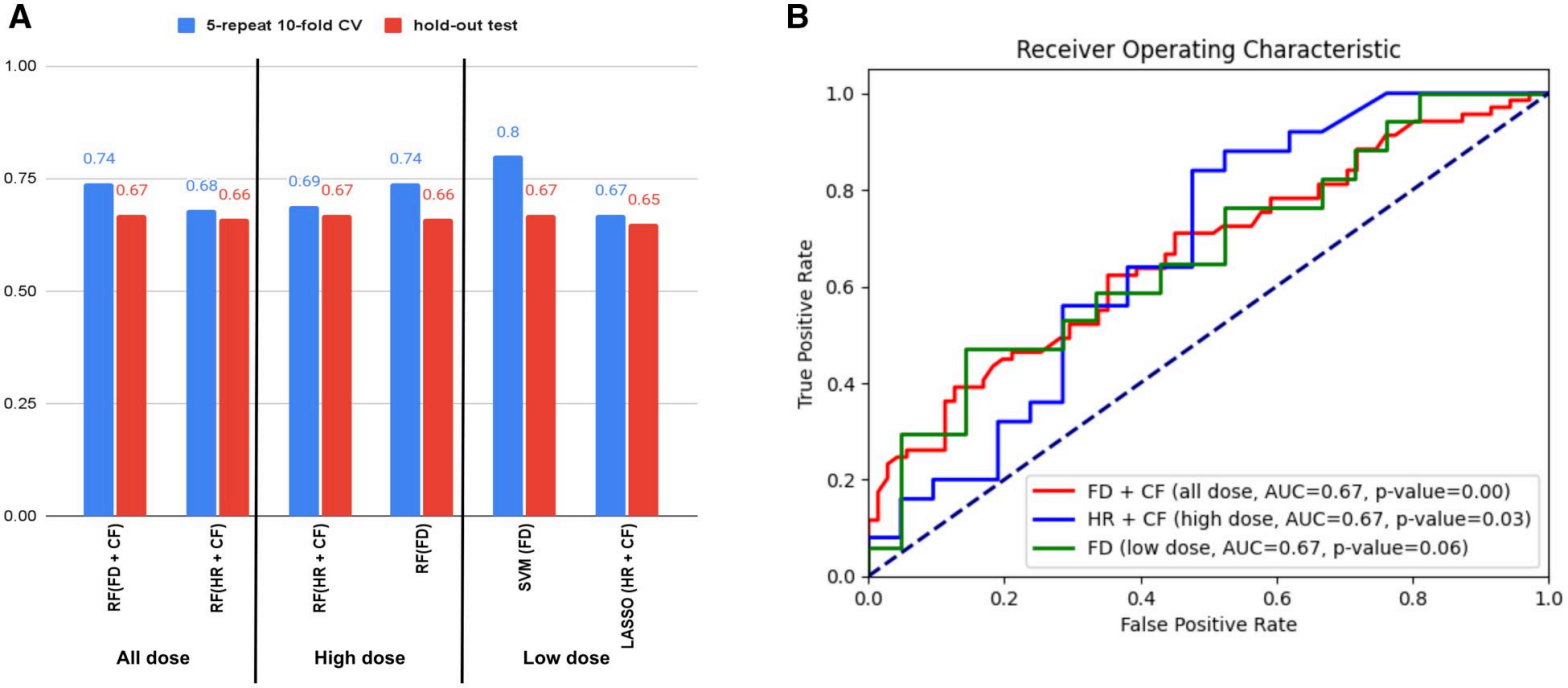
(A) Performance of models based on foundational AI features (FD), hand-crafted radiomics (HR) features, clinical features (CF) and ensembles for the 3 different patient sub-cohorts. Results are shown for the models with the best prognostic performance based on AUC. For the “all dose” and “high dose” cohorts, the RF algorithm yielded highest performance. For the low dose cohort, the support vector machine achieved highest accuracy. Experiments were performed using 5-repeat 10-fold nested cross-validation and are reported for the unseen independent test datasets. (B) The ROC curve for the highest performing model in each arm on an unseen independent test set. RF = random forest; SVM = support vector machine.

Figure 2 illustrates results for the best performing models for each of the sub-cohorts (all dose, high dose, and low dose) derived from the experiments. Detailed results for all models are presented in the Tables S1-S6.

### All dose sub-cohort analysis

For the sub-cohort consisting of all patients, the univariate Cox analysis identified 822 foundational model features (≤0.05) and hazard ratios, HR: 0.14-3.35, 372 radiomics features (≤0.05) and HR: 0.007-13.8 and 26 clinical features (≤0.05) and HR: 0.56-6.26. Among them, 241 foundational features, 25 classical features along with 19 clinical features were identified as independent predictors of survival. These 285 features were subsequently utilized in the feature engineering and dimensionality reduction process to refine and select an optimal subset of features. The ensemble model based on 7 foundational features combined with 4 clinical features (grade 3 esophagitis, PTV volume, ipsilateral lung mean dose, v5 heart, and v5 lung percentage) achieved the highest AUC of 0.67 (95% confidence interval [CI], 0.58-0.76) on independent testing for the RF algorithm. The second highest performing ensemble model based on 4 classical radiomics features (intensity histogram mode, VOIs volume [mesh], surface to volume ratio, intensity- based energy) combined with 9 clinical features (grade 3 esophagitis, PTV volume, v5 and v20 lung percentage, grade 5 toxicity, v5 heart percentage, grade 3 toxicity, chemotherapy, ipsilateral lung mean dose) achieved AUC of 0.66 (95% CI, 0.57-0.75) using the RF method. The model relying solely on 6 clinical features achieved an AUC of 0.67 (95% CI, 0.58- 0.76) on the test dataset using the LASSO algorithm. The models based solely on radiomics or foundational AI produced similar AUC of 0.64 (test dataset) using RF or SVM algorithms. The ensemble model combining foundational AI, radiomics, and clinical features did not enhance the model performance.

### High dose sub-cohort analysis

For the patient sub-cohort treated with high dose, the univariate Cox analysis identified 659 foundational AI features (≤0.05 and HR: 0.13-4.3), 55 radiomics features (≤0.05 and HR: 0.004-12.3), and 18 clinical features (≤0.05 and HR: 0.41-1.5). Among them, 175 foundational AI features, 12 radiomics features, and 10 clinical features, were identified as independent predictors of survival. These 197 features were subsequently utilized in the feature engineering and dimensionality reduction process to refine and select an optimal subset of features. The ensemble model based on one radiomics feature (Intensity-based energy) combined with 4 clinical features (PTV log volume, age, V95 PTV, and V5 lung percentage) achieved the highest AUC of 0.67 (95% CI, 0.50-0.83) on independent testing for the RF technique. The second highest performing model was based on 5 foundation AI features with AUC 0.66 (95% CI, 0.50-0.83) using the RF method. The RF models based solely on radiomics, and clinical features yielded AUC’s of 0.64 (95% CI, 0.45-0.83) and 0.62 (95% CI, 0.45-0.78) on testing datasets, respectively. The ensemble model combining radiomics, foundational AI, and clinical features did not enhance the model performance.

### Low dose sub-cohort analysis

For the patient sub-cohort treated with low dose, the univariate Cox analysis identified 455 foundational AI features (≤0.05 and HR: 0.14-3.35), 165 radiomics features (≤0.05 and HR: 0.002-2.89) and 18 clinical features (≤0.05 and HR: 0.56-6.26). Among them, 170 foundational radiomics features, 19 classical radiomics features along with 19 clinical features, were identified as independent predictors of survival. These 208 features were subsequently utilized in the feature engineering and dimensionality reduction process to refine and select an optimal subset of features. The model solely based on 12 foundational AI features achieved the highest AUC of 0.67 (95% CI, 0.50-0.85) on the testing dataset using the SVM method. The second highest performing ensemble model yielded AUC of 0.65 (95% CI, 0.47-0.83) using the LASSO/GLM method. This model combined 7 radiomics features (maximum histogram gradient intensity, small distance high grey level emphasis, neighborhood grey tone difference-based contrast, surface area [mesh], intensity-based energy, volume fraction difference between intensity fractions [10%-90%], intensity-based coefficient of variation) with 5 clinical features (grade 5 toxicity, PTV log volume, V20 esophagus percentage, heart mean dose, and ipsilateral lung mean dose). The RF model based solely on radiomics or clinical features achieved similar AUC’s of 0.64 (95% CI, 0.42-0.79) and 0.61 (95% CI, 0.42-0.79) in testing, respectively. The ensemble model combining radiomics, foundational AI, and clinical features did not enhance the model performance.

## Discussion

We developed prediction models using significant features from a foundational AI architecture, hand-crafted radiomics, and clinical factors. The foundational AI model and the hand-crafted radiomics model yielded similar results on independent test datasets. The development of ensemble models combining foundational AI, radiomics, and clinical features did not improve performance. However, ensemble modeling with either foundational AI or radiomics combined with clinical factors did marginally improve model performance. Further investigation of the ensemble model approach is needed to understand potential complementary value. This study is among the first to investigate the prognostic and complementary value of foundational radiomics with clinical features and classical radiomics using various popular ML algorithms. Our results were similar to those reported by Pai et al^12^ who used a foundational AI architecture with a single linear classifier.

The modeling methods were performed on the planning CT image datasets prior to radiation being delivered.^18^^,^^19^ The results are therefore suggestive of associations found on inherent signatures in the planning CT datasets not affected by the radiation treatment dose. In this regard, it is interesting but perhaps not surprising to note that AUCs in the different sub-cohorts (all dose, high and low doses) were not significantly different. Similar research, albeit not in the context of foundational AI, was performed by Parmar et al^19^ who extracted radiomics features from planning CT images and conducted feature selection for evaluating prognostic performance for a cohort of head and neck cancer patients. They showed AUC values in the range of 0.6-0.7 for prognostic performance using models including RF and SVM.

Our study has notable limitations. Application of the foundational model was done with minimal fine-tuning based on our patient datasets. We are presently expanding our work to fine tune the foundational model and include additional anatomical structures such as the heart and lung, to characterize normal tissues as well. As with radiomics research, factors such as image intensity normalization, inter-observer segmentation uncertainties, image processing techniques, image filtering methods, and radiomics feature harmonization potentially impact on the selection of statistically significant radiomics features.^15^ We followed the guidance specified by the IBSI^15^ which is imperative toward generating consistent and reproducible radiomics features across multiple cancer types, which can then be reliably connected to outcomes and ultimately produce sets of validated biomarkers for different cancer types. Appropriate sample sizes are necessary to yield optimal results in designing “big data” statistical models. Despite the analysis of several hundred patients in the RTOG 0617 dataset, the extraction of thousands of features suggests that the sample size is likely inadequate. We are currently engaged in research to understand the tradeoffs between the number of features extracted dimensionality reduction in the context of the dataset bias, variance, and generalization error.

## Conclusion

We developed and compared multiple prognostic models based on a self-supervised foundational AI model, hand-crafted radiomics signatures, and clinical factors. These models and combinations (ensemble) of them showed encouraging results for prediction of 2-year OS for patients with locally advanced NSCLC treated on the NRG/RTOG 0617 clinical trial. These models developed from retrospective patient data can be potentially applied to benefit patients treated prospectively. Further investigation is warranted using larger datasets to elicit more conclusive results and to explore the potential complementary value of ensemble modeling.

## Supplementary Material

tzae038_Supplementary_Data
